# Effect of Parental Adverse Childhood Experiences on Intergenerational DNA Methylation Signatures

**DOI:** 10.21203/rs.3.rs-2977515/v1

**Published:** 2023-06-26

**Authors:** Sahra Mohazzab-Hosseinian, Erika Garcia, Joseph Wiemels, Crystal Marconett, Karina Corona, Caitlin Howe, Helen Foley, Deborah Lerner, Nathana Lurvey, Shohreh Farzan, Theresa Bastain, Carrie Breton

**Affiliations:** University of Southern California

## Abstract

Adverse Childhood Experiences (ACEs) are events that occur before a child turns 18 years old that may cause trauma. In this study, the effect of cumulative ACEs experienced on human maternal DNA methylation (DNAm) was estimated while accounting for interaction with domains of ACEs in prenatal peripheral blood mononuclear cell samples from the Maternal and Developmental Risks from Environmental Stressors (MADRES) pregnancy cohort. The intergenerational transmission of ACE-associated DNAm was also explored used paired maternal and neonatal cord blood samples. Replication in buccal samples was explored in the Children’s Health Study (CHS). We used a four-level categorical indicator variable for ACEs exposure: none (0 ACEs), low (1–3 ACEs), moderate (4–6 ACEs), and high (> 6 ACEs). Effects of ACEs on maternal DNAm (N = 240) were estimated using linear models. To evaluate evidence for intergenerational transmission, mediation analysis was used. Analysis of maternal samples displayed some shared but mostly distinct effects of ACEs on DNAm across low, moderate, and high ACEs categories. *CLCN7* and *PTPRN2* was associated with maternal DNAm in the low ACE group and this association replicated in the CHS. ACE-associated methylation was observed in maternal and neonatal profiles in the *COMT* promoter region, with some evidence of mediation by maternal *COMT* methylation. Specific genomic loci exhibited mutually exclusive maternal ACE effects on DNAm in either maternal or neonatal population. There is some evidence for an intergenerational effect of ACEs, supported by shared DNAm signatures in the *COMT* gene across maternal-neonatal paired samples.

## Introduction

Adverse Childhood Experiences (ACEs) refers to a collection of adverse events that occurs before a child turns 18 years old [1]. Accumulating ACEs are associated with a heightened risk of several unfavorable outcomes – including all-cause mortality [2]. ACEs are common, with 57% of adults reporting more than one ACE. Exposure to ACEs can be quantified as an increasing number of events overall, or assessed within domains of abuse (sexual, physical, or emotional), neglect (emotional or physical), and dysfunction (depression, incarceration, domestic violence, substance abuse, and mental illness) [1]. ACEs are also associated with increased rates of violence and victimization. This pattern can manifest multi-generationally: children of individuals who experiences ACEs are at increased risk for experiencing ACEs themselves [3, 4]. Strikingly, this multigenerational effect of ACEs is present from birth: infants born to mothers with high ACEs exhibit aberrant stress responses compared to infants of mothers with no ACEs [5]. Such evidence may speak to larger biological and adaptive processes activated in response to chronic early life stress [6].

Improvement in trauma informed care (TIC) reduces risk of physical and mental health outcomes for individuals exposed to ACEs [7]. Investigating the biological underpinnings of ACEs can aid in these efforts. One biological consequence of ACEs is reflected in DNA methylation changes (DNAm). DNAm is the molecular state captured when a methyl group is covalently bonded to the cytosine base of DNA, potentially influencing gene expression. The effects of early life adversity, maltreatment, and/or ACEs have been studied extensively within human and animal models in relation to DNAm [8–10]. Literature based on animal models is experimental and usually examines the effect of an isolated adverse event. Most human studies have focused on either specific domains or overall number of ACEs and few studies have explored the combinatorial effect of such factors. It is important to consider these two measures in tandem because the magnitude and domain of ACEs may contribute to variability in DNAm in human populations [11].

This study first aimed to examine the effect of the total number of ACEs experienced on maternal peripheral blood mononuclear cell (PBMC) DNAm during pregnancy while accounting for interaction with specific domains of ACEs. The possible intergenerational transmission of ACE-associated DNAm was also explored using paired maternal and child PBMC DNAm profiles in a mediation analysis. Relationships between maternal PBMC DNAm and maternal PBMC gene expression was evaluated. The analysis was conducted in a discovery and replication study from two population-based cohorts in Los Angeles, CA, using two distinct tissue types. Data from the Maternal and Developmental Risks from Environmental and Social Stressors (MADRES) pregnancy cohort [12] using maternal and cord blood PBMC DNAm and gene expression was used as the discovery population and the Children’s Health Study (CHS) using family-based parental adult and pediatric child buccal cell data served as the replication population [13, 14]

## Materials and Methods

### Discovery Study Sample and Recruitment

Recruitment of pregnant people in the MADRES prospective pregnancy cohort began in 2015. Participants were recruited through four prenatal providers in Los Angeles, California if they were less than 30 weeks gestational age at cohort entry, at least 18 years old, and provided informed consent (IRB# HS-15-00498). Additional exclusion criteria include incarceration during recruitment, multiple pregnancy, and HIV-positive status. Pregnant people were interviewed at each trimester, birth, and postnatally for up to a year. Details on all variables collected and additional exclusion criteria in the MADRES study is available here [12].

### MADRES Maternal DNAm

PBMCs were isolated from 10 mL peripheral blood samples collected during the early and late pregnancy visit (Figure S1). DNA was extracted using the AllPrep DNA kit (Qiagen). Samples were stored within one hour of collection at −80°C. Bisulfite conversion of DNA was conducted using the EZ DNAm Kit (Zymo Research). Then, DNAm was quantified using the Illumina Infinium HumanMethylationEPIC assay using the manufacturer’s recommended protocol with no other modifications.

### MADRES Cord Blood DNAm

10 mL of umbilical cord blood was collected at birth and stored at −80°C within 24 hours of delivery. PBMCs were isolated from the cord blood, and DNA was extracted, and bisulfite converted using the EZ DNAm Kit (Zymo Research). The Illumina Infinium HumanMethylationEPIC was used to quantify DNAm under the same protocols as the maternal arrays.

### MADRES DNAm Quality Control

All data analysis was performed in R (version v4.1.0, R Core Team 2021). Quality control and normalization of data were performed separately for maternal and cord blood samples. Sample and probe level quality control were performed using standard protocols outlined by the minfi Bioconductor package [15]. Briefly, poor detection p-values were computed across probes, representing those probes with no significant difference in detection between background and control probes, and were removed from the analysis. If a sample had more than 10% of poor (p > 0.01) detection p-value probes, it was removed from the analysis. Cross-reactive and polymorphic probes were also removed [16–17]. Outlier individuals, or those displaying a median probe intensity below the minfi default value of 10.5, were also removed from the analysis. Sex predicted from intensity of X and Y chromosomes was used as a quality control check. Noob background correction for dye-bias followed by quantile normalization was used from for normalization. SNP-associated probes were removed from the analysis. Log-transformed beta-values were used in downstream regression analysis [15]. Figure S2 is a consort diagram of our quality control and sample normalization process. In the maternal samples, 5% of samples failed quality control. In cord blood samples, 15% were dropped due to low median intensities (N = 16) and sex discrepancy (N = 3).

### MADRES Maternal RNA-seq

Maternal PBMC gene expression levels were profiled for the same mothers with DNAm profiles in pregnancy. PBMCs were isolated from 10 mL of whole blood collected in early pregnancy (N = 50). Total RNA was extracted and sequenced using Illumina HiSeq4000 by the Dartmouth Genomics Core Laboratory following a standard protocol. Samples displayed good quality with a depth between 20 and 70 million reads for each sample with a median alignment rate of 85%. MultiQC plots were generated to examine sample quality across maternal profiles – no samples were flagged for removal from the analysis based on phred score, per base N content, or per sequence GC content [18]. The HISAT2 aligner was ued to map reads to the reference; GRCh genome annotation number 97 from Ensembl [19–20]. FeatureCounts was used to quantify reads to exons [21]. Transcripts per million (TPM) log2-transformed counts were generated from raw counts in downstream correlative analysis [22]. Our sample was limited to participants with available ACE questionnaire and DNAm data (N = 35). These data are publicly available in Gene Omnibus Expression (Accession Number: GSE18175).

### Children’s Health Study DNAm

The CHS is a prospective cohort study that recruited schoolchildren from the Southern California region from the 1990s to 2000s [13–14]. The study’s goal was to determine the effects of air pollution and other environmental exposures on respiratory health. Buccal cell samples were collected from a subset of recruited participants. Approximately two decades after baseline, a convenience sample of adult-aged index participants were invited to participate in a follow-up mail-based study. Buccal samples were collected from the adult participant, their partner, and their child. Informed consent was provided (IRB #HS-17-00778). Pediatric buccal cells were collected with swab using the Oragene OC-175 kit or toothbrush collection methods for each family trio in approximately 5mL of buffer. DNA was extracted with the Oragene prepIT L2P kit. The extracted DNA was stored locally at −80°C. The Zymo EZ DNAm kit was used to perform bisulfite conversion, and the Illumina EPIC DNAm protocol was used to generate the data. For the purposes of replication, the CHS samples was limited to the recently collected adult (N = 31) and offspring pediatric (N = 173) samples. All sample and probe level filters applied to the discovery population were applied to the replication population in the CHS. Figure S3 is a consort diagram for the pediatric and parental populations. 12% of our samples and 90% of probes were retained from the parental population, while 67% of samples and 90% of probes were retained in the pediatric participants.

#### *Cell Type* Estimation

Cell type estimation was conducted separately on discovery and replication datasets. The Houseman method using the EPIC reference platform was executed in minfi [15] using the FlowSorted.Blood.EPIC data for cord blood and whole blood, respectively [23]. Cord blood and blood composite cell type was used for neonatal and maternal data respectively in MADRES. For buccal cell data in the CHS, immune cell type proportions were estimated using HEpiDISH [24].

### Genetic Factors

Given the confounding impact of ancestral based SNP-variation on DNAm in this analysis, we used EPISTRUCTURE principal components generated from the quality-controlled DNAm dataset. The top two EPISTRUCTURE principal components were used in maternal and neonatal analyses. EPISTRUCTURE methods are described elsewhere. Briefly, the program uses sparse principal component analysis and was validated in multi-ethnic populations [25].

### Measurement of Adverse Childhood Experiences

The Adverse Childhood Experiences (ACEs) CDC-Kaiser questionnaire is shown in Figure S4 [1]. The ACEs questionnaire was administered during the second pregnancy trimester in MADRES. In CHS, the questionnaires were distributed during the mail-based follow-up and were administered to both parents and children. The same questionnaire was administered in both the discovery and replication populations. ACE scores were treated as a categorical indicator variable for all analyses: none (0 ACEs), low (1–3 ACEs), moderate (4–6 ACEs), and high (> 6 ACEs).

### Covariate Selection

In addition to cell type, batch, gestational age at sample collection [26–27], and genetic covariates identified above, confounders were identified a priori from the literature (Figure C1, Supplemental Text). For estimating maternal effects, the following covariates were included: maternal age at birth, pre-pregnancy body mass index (BMI), a composite variable for National Institute of Health (NIH) race categories, ethnicity, and nativity status (Black and Non-Hispanic, Foreign-born Hispanic, Multiracial, US Born Hispanic, and White Non-Hispanic), any evidence of glucose dysregulation, any evidence of a hypertensive disorder, socioeconomic status defined as highest maternal education attained (less than high school, high school or some college, and any college degree), nulliparity status (binary defined as first pregnancy), prenatal smoking use (binary defined as mothers who reported smoking in both their early and late pregnancy visits), and maternal depressive symptoms using the Center for Epidemiology Studies for Depression (CES-D) Scale (cut-off of 16 for probable clinical depression) [28]. In addition, the top two surrogate variables produced from surrogate variable analysis (SVA) was used to control for technical variation in our maternal and neonatal datasets. SVA uses probe level data to estimate unwanted sources of heterogeneity and produces variables that capture the variability in said heterogeneity [29]. The replication population adjusted for the same covariates, including sex in the parental population. Depressive symptoms were also measured in binary questionnaire (have you ever been diagnosed with depression by a doctor?) in CHS compared to MADRES. The pediatric CHS model also included a covariate for ACE score of the child. Technical covariates were not adjusted for in mediation analysis. Additional information on covariate selection can be found in the supplemental text.

### Statistical Analysis

The objective was to estimate effects of ACEs on DNAm and then determine if these effects show evidence of intergenerational transmission using a mediation analysis.

Effects of each ACE category on maternal DNAm was estimated by adjusting for confounders detailed above. Linear regression models were implemented in limma [30] accounting for correlation between the early and late pregnancy visit (n = 120, N = 240) in our prenatal samples (Figure S1) to estimate differentially methylation positions (DMPs) and differentially methylated regions (DMRs) [31]. Interactions with domains of abuse and neglect were analyzed within the low and moderate ACE categories because individuals in the high ACE group all had experiences across the three domains. A similar approach for the main effect was used in the neonatal cord blood dataset. Then, using the average beta value among the probes mapped to maternal DMRs annotated to RNA-sequencing genes, Pearson correlation were used to measure the direction and strength of the associations between maternal DNAm and TPM-normalization maternal expression of the associated gene during pregnancy.

Second, overlapping significant (FDR < 0.05) DMRs between maternal and neonatal datasets were used as candidates for the intergenerational mediation analysis. Significance of the indirect pathway was tested using a quasi-Bayesian method for p-value and confidence interval estimation from the mediation package [32]. Mothers reporting persistent prenatal smoking (n = 3) were removed from the mediation analysis due to potential for exposure-induced mediator-outcome confounding, resulting in a total of 60 mother-child pairs (Figure S2). Additional statistical models for mediation sensitivity analysis are described in the supplemental text.

## Results

### Characteristics of the Study Populations

Characteristics of MADRES participants (N = 240, n = 120) are presented in Table 1. Individuals with higher ACE scores were more likely to have lower educational attainment (p = 7.88 × 10^− 4^). Prevalence of ACEs also varied significantly by race, ethnicity, and foreign-born status (p = = 7.23 × 10^− 8^). Individuals also differed in their diabetes prevalence (p = 0.0112), with a lower prevalence in the low ACE group. Characteristics of CHS index adult subjects (n = 31) and pediatric participants (n = 173) are listed in Table S1 and Table S2.

### Associations between ACEs and Maternal DMPs/DMRs

**Figure 1:**
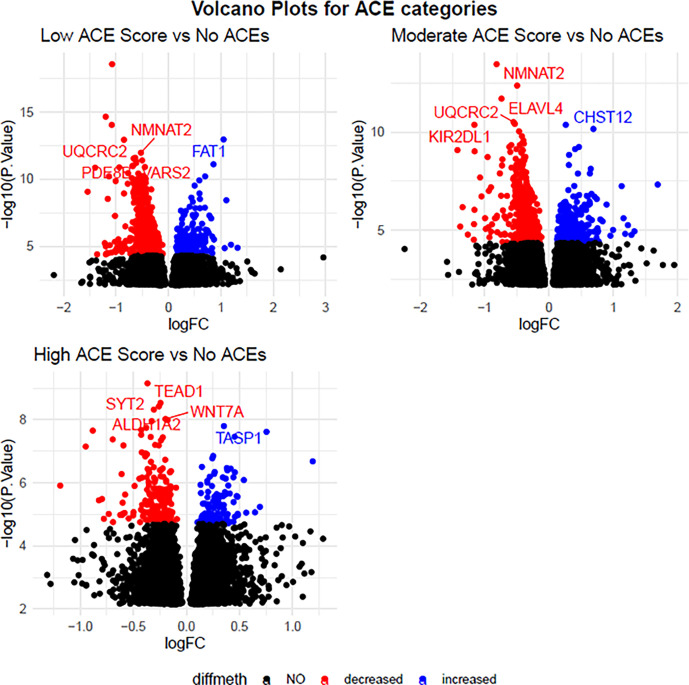
Volcano Plots for the Effect of ACEs on Maternal DNA methylation Top five p-value significant (FDR < 0.05) annotated genes for each ACEs category: none (0 ACEs), low (1–3 ACEs), moderate (4–6 ACEs), and high (>6 ACEs) are labelled. All red and blue dots are significant probes (FDR < 0.05), all black dots are those that did not meet FDR significance (FDR > 0.05). Blue indicates a log-fold change with methylation increased in ACE groups versus the control group, and red indicates a log-fold change with methylation decreased in exposed versus unexposed individuals.

**Figure 2:**
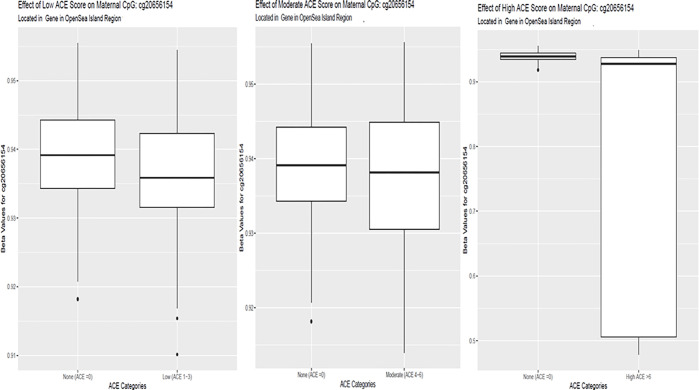
Boxplots of Stable ACE effects in Significant Maternal Probes Boxplot of beta values for Maternal CpG cg20656154 significant (FDR < 0.05) across higher ACE scores. There was a trend for decreased methylation in higher ACE scores compared to those with no ACEs.

In a DMP analysis using individuals with no ACEs as the reference, we identified 784 DMPs associated with a low ACE score, 710 DMPs associated with a moderate ACE score, and 313 DMPs associated with a high ACE score (Fig. 1). 67%, 61% and 62% of DMPs had decreased methylation in low, moderate, and high ACE scores compared to none. In a DMR analysis using individuals with no ACEs as the reference, we identified 138 DMRs associated with low ACE score, 133 with a moderate ACE score, and 56 with a high ACE. A table of the top 10 DMRs is listed in Table 2. Two genes and 10 DMPs (Fig. 2) were consistently identified as having DMRs across the ACE categories: *COMT/TXNRD2* and *KCNQ1*.

### DMR Interactions by ACE Domain of Abuse and Neglect

49 DMRs were associated with abuse in the low ACE category, and 166 in the moderate group. Across the low and moderate group, the following DMRs were consistently identified for the following genes: *LINC01044*, *CFDP1*, *CYP1B1*, *CERS3*, *PRKXP1*, *PPAPDC3*, *EIF2AK4*, *CCDC9*, *C6orf25*.

Exposure to neglect was associated with 68 DMRs among the low ACE group and 141 DMRs in the moderately exposed group. Among individuals with a low or moderate ACE score with experiences of neglect, the following DMRs were shared: *ABAT*, *MBP*, *C2CD2L*, *HLA-DPB2*, *FRMD4A*, *MSL3P1*, *DLEU7*. The top five DMRs for each ACE group and domain are listed in Table 3.

### Effect of ACEs on Neonatal DMPs/DMRs

Among 69 neonates with available cord blood data paired to maternal ACEs, 8 significant DMPs were associated with maternal ACEs in the low category, 2 in the moderate category, and none in the high category. 1 DMR med FDR < 0.05 criteria for significance in the low ACE group (*COMT*) and 2 DMRs were statistically significant in the moderate ACE group (*COMT* and *ZFP57*)

### Intergenerational Mediation Analysis

**Figure 3:**
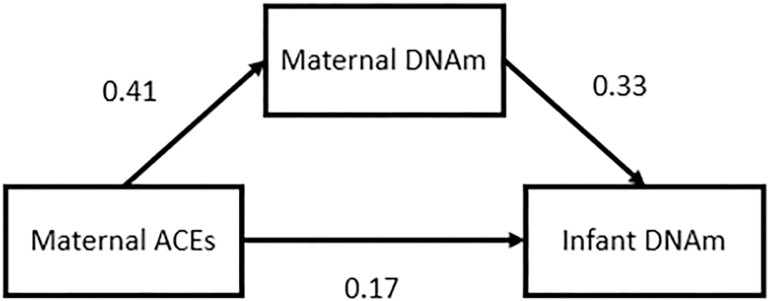
Indirect Effect of ACEs on Intergenerational DNA methylation Signatures Effect of Maternal ACEs on neonatal COMT methylation is partially (64%) mediated by maternal COMT methylation. This indirect effect was significant (p-value = 0.044) with a 95% CI of (0.0025,0.32)

We tested for mediation of the indirect effect of ACEs on neonatal DNAm by maternal DNAm for *COMT/TXNRD2*, a gene with a statistically significant (FDR < 0.05) DMRs identified independently in both maternal and neonatal samples. *COMT* was the only statistically significant (FDR < 0.05) DMR identified across all three ACE categories in prenatal samples and both the low and moderate ACE group among neonatal samples. *COMT* regional DNAm was partially (63%) mediated by maternal DNAm comparing individuals who were exposed to ACEs versus those who were not. The indirect effect was 0.14 with a 95% CI of (0.0025, 0.32) (p-value = 0.044) (Fig. 3).

### Correlation between Maternal Methylation in DMRs and Gene Expression

Among 144 statistically significant low ACE DMRs mapped to HCNC gene names, 13 displayed a nominally significant Pearson correlation (p < 0.05) with gene expression in the first trimester (Table S3). These included: *NMNAT2*, *HDAC4*, *GAK*, *MFAP3*, *VARS2*, *MAFK*, *PON1*, *PTPRN2*, *LYNX1*, *TUBGCP5*, *CLCN7*, *GPR108*, *AND TXNRD2*.

### Replication Cohort: ACE-associated Adult DMRs

In our evaluation of ACEs and adult DNAm in the adult replication cohort, a total of 82 DMRs were found in the low ACE group, 60 in the moderate ACE group, and six in the high ACE group. We replicated 2 DMRs from the low ACEs group and 1 DMRs from the moderate ACEs group in the CHS at an FDR < 0.05. Among the low ACE group DMRs, the *CLCN7* and *PTPRN2* gene were replicated (Figure S5). DMRs in both genes were statistically significant in the moderate ACE group in CHS. Among the moderate ACE groups, the *FOXK1* replicated across CHS and MADRES. There were no shared DMRs across the high ACE groups. Effect sizes across overlapping statistically significant DMRs were consistent in direction for both cohorts. When comparing the direction of effect among probes identified (FDR < 0.05) in MADRES prenatal samples, approximately 50% of probes were consistent in the same direction in our replication population (Figure S6).

### Replication Cohort: Parental ACE Intergenerational Pediatric DMRs

A total of 53 DMRs were found in the low ACE group, 36 in the moderate ACE group, and five in the high group. We replicated no DMRs from the neonatal DMRs in MADRES evaluated at an FDR < 0.05. Among the maternal MADRES genes, the *GAK* was replicated in the low ACE group. The replicated *GAK* DMR overlapped and were consistent in direction for the two cohorts.

### Gene Ontology Analysis

To capture shared pathways influenced by exposure to ACEs, we input all unique DMP-associated genes to ShinyGo [33]. This was done independently for the maternal discovery and adult replication cohorts to determine potential overlap. Gene ontology analysis of the discovery population indicated the top pathways to be enriched for KEGG processes in nicotine addiction, GABAergic synapse, and oxytocin signaling pathway (Fig. 5). For the replication population, there were no enriched KEGG pathways.

## Discussion

Few studies have explored shared signatures of childhood adversity on an epigenome-wide scale in multiple generations. Prior studies have generally been limited to candidate probe or regional studies in a single generation. Specifically, there has been focus on candidate genes including *BDNF*, *NR3C1*, *AVP*, *and FKBP5* [9]. No DMRs in the main effect or interaction model demonstrated a statistically significant (FDR < 0.05) overlap with these candidate genes. However, there was overlap in our main effect and interaction models with other studies using genome-wide scans, including *CACNA2D4* [34], *ALS2* [35], *OPRL1* [36], *PRDM16*, and *C8orf31* [10]. *ZFP57* was statistically significant in the interaction of abuse in the maternal population (Table 3) and the main effect in our neonatal population and has been associated with a shared multigenerational signature of neglect in human studies [37]. Moreover, *CLCN7* and *PTPRN2* were two overlapping statistically significant DMRs in our low ACE group in the discovery and replication population. *PTPRN2* has been implicated with exposure to stress, violence and associated with mood state [38–39]. *CLCN7* is associated with osteoporosis [40]

**Figure 4:**
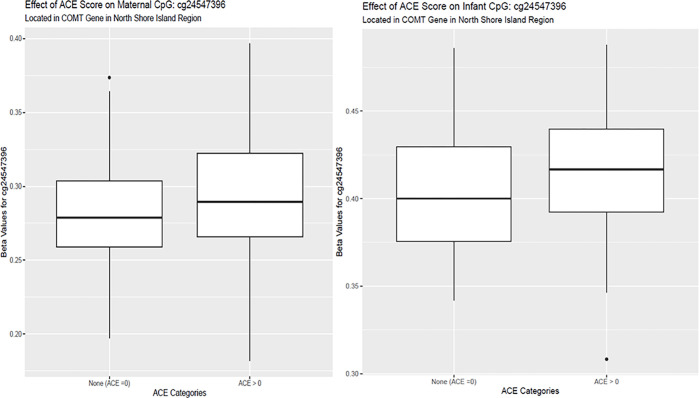
Boxplots of *COMT* Effect in Maternal and Neonatal Samples Beta Values for COMT regional methylation in maternal and neonatal samples in MADRES.

**Figure 5:**
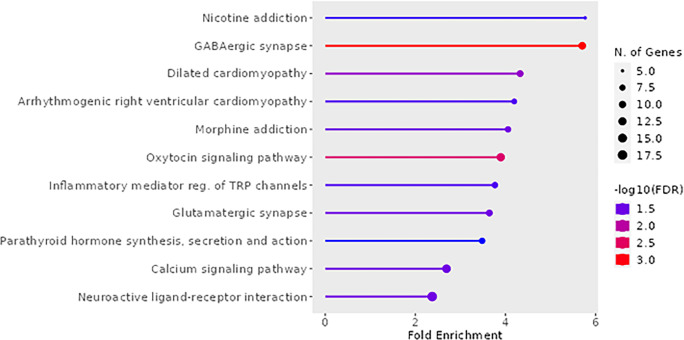
KEGG pathways in ACE-associated DMPs in Maternal Discovery Population Significant (FDR < 0.05) maternal DMP genes in MADRES input to ShinyGO to generate KEGG pathways

We identified a DMR in the *COMT/TXNRD2* gene for both mothers and neonates. We therefore tested whether maternal methylation levels in this gene mediated ACE impacts on neonatal methylation levels. *COMT/TXNRD2* met the criteria for intergenerational mediation of ACEs on neonatal methylation levels. The DMR was identified in the north shore and island region of the *COMT* gene, overlapping with the end of the *TXNRD2* gene and located in the 22q11.2 region [41]. This gene is involved in dopamine metabolism and methylation of this gene is associated with stress, pain sensitivity, and diagnosis of several neurological outcomes in adults [42]. Increased *COMT* methylation has been associated with malnutrition and impaired cognition, consistent in the direction of our adult discovery cohort [43]. Increasing promoter methylation of *COMT* has been associated with an increased risk of experiencing stress, consistent with the direction in our adult discovery cohort [44] (Fig. 4). Although *COMT* promoter methylation has been associated with Val158 polymorphism [45] twin studies indicate that there is residual variation not explained by genotype [46–47]. The *COMT* Val158 polymorphism has a frequency of approximately 40% in Latin American populations [48], and therefore has the potential to bias our results away from the null. Therefore, even with control for EPISTRUCTURE genetic ancestry principal components, we cannot rule out the possibility of residual confounding by genotype. This intergenerational effect in the discovery population did not replication, and the *COMT/TXNRD2* gene was not statistically significant in either adult or pediatric participants.

The lack of replication for some of our findings between MADRES and the CHS may reflect differences in tissue types, sex distributions, race, ethnicity, foreign born status, or age differences between the two cohorts. Our discovery population also had a higher prevalence of moderate and high ACE score exposure. However, there was also a higher prevalence of college educated individuals in our replication population, which may indicate that the replication population had access to resources to mitigate effects of ACEs, effectively biasing results towards the null. Moreover, the replication population was a convenience sample from the original cohort. It is therefore possible that selection bias may be distorting our results.

This study used a DAG to guide covariate selection. We adjusted for confounders to promote exchangeability in our analysis. However, assumptions of consistency of the effect of ACEs on DNAm may be violated given that effects of time and circumstance may render shared experience among individuals with a similar ACE score. With regards to mediation analysis, assumptions of exchangeability are met. Exposure-induced mediator outcome confounding may be present due to maternal adulthood trauma, adulthood socioeconomic status, and maternal BMI. Maternal BMI was tested for evidence of exposure-induced mediator outcome confounding using a multiple sequential mediators’ product method [49] and adult socioeconomic status was restricted to the most prevalence group in our sample. Effects of maternal adulthood trauma were unmeasured in our cohort, so these may be conflating estimates. Additional discussion on DAG pathways and mediation sensitivity analysis can be found in the supplemental text.

Our study is one of the first to demonstrate some evidence of an intergenerational effect on an epigenome-wide scale with regards to early childhood adversity. One strength of the study was the rich discovery dataset, which enabled us to test for effects of ACEs on DNAm. However, the sample size in our discovery population was modest and may have been subject to Type I error. We were also limited in the overlap of findings for our replication cohort.

In this study, we observed that higher ACE scores were associated with DNAm both in our discovery prenatal sample and adult replication population. While some effects were conserved across low, medium, and high ACE scores, most effects were distinct across ACE categories and domains. More DMRs were shared between low and moderately exposed ACE categories than low and high exposed ACE categories, in part possibly due to higher statistical power.

Overall, two FDR-significant DMRs in our prenatal discovery population, *PTPRN2* and *CLCN7*, replicated in our adult buccal cell population. *CLCN7* was also statistically significant in the gene expression correlation analysis in the discovery population. Among the neonatal cord blood discovery and pediatric buccal replication populations, the *GAK* gene was replicated. A majority of identified DMRs and DMPs in the discovery and replication population were mapped to Open Sea regions.

The *COMT/TXNRD2* DMR demonstrated an intergenerational association in our discovery population, but this effect did not persist in the replication analysis. Moreover, in our adult discovery population, methylation of this DMR was positively associated with gene expression.

## Figures and Tables

**Table 1:** Baseline Characteristics of Analytic Discovery Sample (N = 120)

		No ACE ACE 1–3	ACE 4–6	ACE > 6	P-Value^A^
	41(34)	55(46)	19(16)	5(4)	
Maternal Age at Birth					
Age(SD)	29.0(5.4)	28.9(6.2)	30.2(5.6)	29.1(6.6)	0.664
**Smoking Status N(%)**					
Persistent Smoking^B^	1(2)	2(4)	0(0)	0(0)	0.612
**Pre-Pregnancy BMI** ^C^					
Mean(SD)	28.3(4.9)	27.9(6.9)	28.9(8.1)	28.5(6.3)	0.875
**CES-D** ^D^ **Score**					
Depression	9(22)	11 (20)	5(26)	2(40)	0.479
**Perceived Stress Score** ^E^					
High Stress	0(0)	2(3.6)	0(0)	0(0)	0.497
**Diabetes Status N(%)**					
Insulin disorder	19(46)	13(24)	7(37)	2(40)	0.0112
**Hypertensive Status N(%)**					
Hypertensive disorder	13(32)	11 (20)	3(16)	1(20)	0.160
**Race/Ethnicity N(%)**					7.23 × 10^− 8^
Black,Non-Hispanic	2(4.8)	12(22)	0(0)	0(0)	
Hispanic, Foreign-Born	8(20)	16(29)	9(47)	0(0)	
Hispanic, Non-Foreign	27(65)	23(42)	5(26)	4(80)	
Multiracial	1(2.4)	0(0)	2(10)	0(0)	
White,Non-Hispanic	3(7.3)	4(7.3)	3(16)	1(20)	
**Maternal Education (N%)**					7.88 × 10^− 4^
Less than High School	14(34)	12(22)	2(10)	3(60)	
High School Degree or some college	22(54)	34(62)	10(52)	1(20)	
Any College Degree	5(12)	9(16)	7(37)	1(20)	
**Parity Status N(%)**					
First child?	29(71)	32(78)	11 (58)	3(60)	0.305
**Gestational Age at Birth**					
Mean(SD)	38.8(1.5)	39.1(1.4)	39.5(1.3)	39.5(0.9)	0.393

A Unadjusted p-values derived via chi-square tests for categorical variables and one-way ANOVAs for continuous variables

B Defined as individuals who reported smoking at early and late trimester

C Body Mass Index (BMI)

D Center for epidemiologic scale for depression at second trimester visit, cut-off of 16 used for probable depression

E Perceived Stress Score at second trimester visit, high stress characterized as a score > 27

Top five p-value significant (FDR < 0.05) annotated genes for each ACEs category: none (0 ACEs), low (1–3 ACEs), moderate (4–6 ACEs), and high (> 6 ACEs) are labelled. All red and blue dots are significant probes (FDR < 0.05), all black dots are those that did not meet FDR significance (FDR > 0.05). Blue indicates a log-fold change with methylation increased in ACE groups versus the control group, and red indicates a log-fold change with methylation decreased in exposed versus unexposed individuals.

**Table 2:** Top 10 DMR’s Average Regional Beta Value Difference (ARBVD) in ACE categories

ARBVD	FDR	Gene Name	Chr	Start	End	No.CpGs
Low						
−0.12	3.6e-26	N/A	chr18	77376639	77377589	5
−0.10	2.7e-06	N/A	chr8	49427275	49427415	3
−0.09	7.3e-08	PTPRN2	chr7	157369895	157369960	3
0.09	5.8e-06	N/A	chr9	100881931	100881995	2
−0.09	4.7e-10	PON1	chr7	94953653	94954202	8
0.09	7.6e-06	N/A	chr10	101282726	101282883	4
0.09	7.2e-08	MPRIP	chr17	17109640	17109817	7
−0.08	5.5e-07	AC01657.3	chr2	239140032	239140318	5
0.08	4.9e-10	MAFK	chr7	1572252	1572327	2
0.08	1.5e-05	HDAC4	chr2	240241154	240241218	2
Moderate						
0.13	3.9e-05	CYP4V2	chr4	187125958	187126073	2
−0.12	6.2e-09	N/A	chr6	36639949	36640019	2
−0.11	1.8e-05	N/A	chr8	49427275	49427415	3
0.10	1.1 e-11	AC006033.22	chr7	38350921	38351226	5
−0.09	9.1e-09	N/A	chr18	77377119	77377589	3
−0.09	2.6e-18	KIR3DL1	chr19	55280672	55281274	5
−0.08	1.7e-11	KCNN3	chr1	154839909	154839983	2
−0.08	2.7e-05	PON1	chr7	94953653	94954202	8
0.07	1.4e-06	N/A	chrX	8751330	8751402	2
0.07	2.6e-06	FMOD	chr1	203320506	203320541	2
High						
0.11	1.7e-06	NA	chr15	56299380	56299382	2
−0.09	1.7e-05	LINC01044	chr13	112978683	112978703	2
−0.08	1.8e-09	KCNN3	chr1	154839813	154839983	3
0.08	6.5e-06	FLG-AS1	chr1	152161885	152161927	2
−0.06	1.0e-07	BAIAP2L1	chr7	98029266	98029285	3
−0.06	2.8e-09	NA	chr2	88469730	88469819	4
−0.06	4.8e-12	FAM194A	chr3	150421255	150421424	3
0.06	6.6e-06	NA	chrX	8751266	8751402	3
0.05	6.5e-06	NA	chr6	28853021	28853050	2
−0.05	7.3e-08	HLA-C	chr6	31239243	31239411	5

The top ten highest magnitude of ARBVD significant DMRs displayed in each of the ACE categories. The ARBVD is a region defined by DMRcate() as a group of CpGs (> 1) with a stable beta value difference between each of the ACE groups and control, adjusted for the variables in our linear model.

Boxplot of beta values for Maternal CpG cg20656154 significant (FDR < 0.05) across higher ACE scores. There was a trend for decreased methylation in higher ACE scores compared to those with no ACEs.

**Table 3:** Top 10 logFC FDR < 0.05 in ACE domain by ACE category

Mean Difference	FDR	Gene
Abuse		
ACE_1 – 3_		
−0.65	8.4e-11	AKAP13
−0.34	8.7e-08	FMOD
−0.33	3.2e-09	GCSAML
−0.30	1.8e-06	UPK1B
0.27	6.6e-07	HOXB-AS3
ACE_4 – 6_		
0.74	7.0e-13	ZFP57
−0.55	1.8e-22	MOG
0.52	3.2e-33	NA
0.48	2.2e-22	NA
−0.45	2.4e-08	HLA-K
**Neglect**		
ACE_1 – 3_		
0.55	8.2e-09	SLC6A12
−0.35	1.3e-05	RP11
0.34	1.5e-18	Z95704.5
−0.34	7.1e-11	NA
−0.33	7.1e-11	MPRIP
ACE_4 – 6_		
0.86	1.7e-07	HOOK2
0.46	1.9e-12	NA
0.42	4.4e-09	NA
−0.39	3.6e-10	RTEL1
0.36	8.1e-13	TMEM204

Top 10 magnitude of ARBVD for each ACE domain within each ACE score category (interaction).

Effect of Maternal ACEs on neonatal COMT methylation is partially (64%) mediated by maternal COMT methylation. This indirect effect was significant (p-value = 0.044) with a 95% CI of (0.0025,0.32)

Beta Values for COMT regional methylation in maternal and neonatal samples in MADRES.

Significant (FDR < 0.05) maternal DMP genes in MADRES input to ShinyGO to generate KEGG pathways
